# Bilateral Tubo-Ovarian Abscess Colonized by Enterococcus in a Woman With Systemic Lupus Erythematosus and End-Stage Renal Disease: A Case Report

**DOI:** 10.7759/cureus.29631

**Published:** 2022-09-26

**Authors:** Feryal O Attiah, Nada A AlKhateeb, Sara A Marzook

**Affiliations:** 1 Medicine, King Abdulaziz University Hospital, Jeddah, SAU; 2 Urogynecology, King Abdulaziz University Hospital, Jeddah, SAU

**Keywords:** bilateral, esrd, toa, sle, pelvic abscess

## Abstract

Tubo-ovarian abscess (TOA) is an inflammatory process involving the fallopian tubes and the ovary. It is one of the complications occurring in acute pelvic inflammatory disease (PID). Intrauterine device (IUD) insertion, history of a prior PID, reproductive age, and multiple sexual partners are the most common risk factors for TOA. The diagnosis of bilateral TOA is rare. Commonly isolated organisms from the abscess include *Neisseria* (*N.) gonorrhoeae*, *Escherichia​​​​​​*​ (*E.) coli*, and/or normal flora of the vagina and cervix. Treatment of the abscess should be started as soon as possible with broad-spectrum antibiotics as recommended by the Centers for Disease Control and Prevention (CDC) and drainage of the pelvic collection.

Here, we present a complicated case in a middle-aged, sexually inactive woman with bilateral TOA colonized by *Enterococcus faecalis*. She is a known case of systemic lupus erythematosus (SLE) complicated by end-stage renal disease (ESRD), along with other co-morbidities. We summarize, in our case report, the experience of a successful treatment for this condition.

## Introduction

Tubo-ovarian abscess (TOA) is an inflammatory mass involving the fallopian tubes, ovary, and occasionally, other adjacent pelvic organs (e.g., bowel, bladder) [1]. It is one of the serious complications occurring from an acute pelvic inflammatory disease (PID), which contributes to the spread of multiple bacteria from the lower genital tract thereafter, ascending through the uterus (mainly sexually transmitted diseases) to the adjacent structures [2]. Predisposing factors for TOA are like those of PID and include intrauterine device (IUD) insertion, history of a prior PID, reproductive age, and multiple sexual partners [2].

TOA typically occurs in young and sexually active women who present with abdominal pain, pelvic mass, fever, and vaginal discharge [3]. Due to the vague presentation, the differential diagnosis often includes appendicitis, ovarian torsion, ectopic pregnancy, PID, ruptured ovarian cyst, inflammatory bowel disease, diverticulitis, pyelonephritis, and cystitis [2]. Commonly isolated organisms from the abscess include *Neisseria (E.) gonorrhoeae*, *Chlamydia*, *Escherichia (E.) coli*, and/or normal flora of the vagina and cervix [4].

## Case presentation

This was a 38-year-old, single Yemeni lady, who was not sexually active, a known case of SLE complicated by lupus nephritis on prednisone 10 mg daily and 15 mg every other day. Also, she developed ESRD secondary to SLE on regular hemodialysis through a right-sided arteriovenous fistula (AVF) three times weekly and a history of brachial artery thrombosis on clopidogrel 75 mg. Her other chronic issues include hypertension on bisoprolol 2.5 mg, hypothyroidism on 100 mcg levothyroxine, and endometriosis.

She presented to King Abdulaziz University Hospital emergency department complaining of lower abdominal pain for two days. She was in her usual state of health until two days before the presentation, when she started experiencing an acute progressive continuous bilateral lower abdominal pain more on the right side, not radiating, colicky in nature, with no aggravating or alleviating factors.

The pain was associated with a fever of 38.1 ℃, rigors, loss of appetite, vomiting three times (small in amount with no blood), a single episode of loose bowel motion, and grade two shortness of breath. There was no history of weight loss, night sweats, vaginal discharge, vaginal bleeding, dysuria, hematuria, abdominal distension, constipation, jaundice, cough, palpitation, or chest pain.

Upon examination in the emergency department, the patient was looking ill with acute pain, and febrile with a temperature of 39.4 ℃, a pulse rate of 100 beats per minute, blood pressure 102/69 mmHg, and oxygen saturation of 97% in room air. On abdominal exam, there was abdominal guarding, moderate tenderness over the right lower quadrant, mild tenderness over the left lower quadrant, abdominal distention, and an irregular hard mass felt at the lower segment of the abdomen. A vaginal examination was not performed due to the patient's refusal, as she was still a virgin. The rest of the examination was unremarkable.

Bedside transabdominal pelvic ultrasound was performed and showed a cystic structure on the right side, indicating a possible ovarian cyst. The modality was not comprehensive due to the clinical status of the patient, being in pain and dehydrated with a near-empty bladder. On complete blood count (CBC), the patient had a picture of normocytic hypochromic anemia with a hemoglobin of 9.7 (g/dl), leukocytosis of 24.7 (K/uL) with a neutrophil predominance of 23.02 (K/uL), and mild thrombocytopenia of 139 (K/uL). Creatinine was 810 (umol/l), which was above the patient’s baseline, and urea of 15 (mmol/L). The liver function test was unremarkable. INR was slightly prolonged at 1.43, with a normal PTT. Amylase level was elevated at 160 (u/L), with normal lipase. Venous blood gas analysis showed a high lactate level of 3.2 (mmol/L), and high potassium of 5.9 (mmol/L). Bacterial and fungal aerobic and anaerobic blood cultures were all negative.

**Figure 1:**
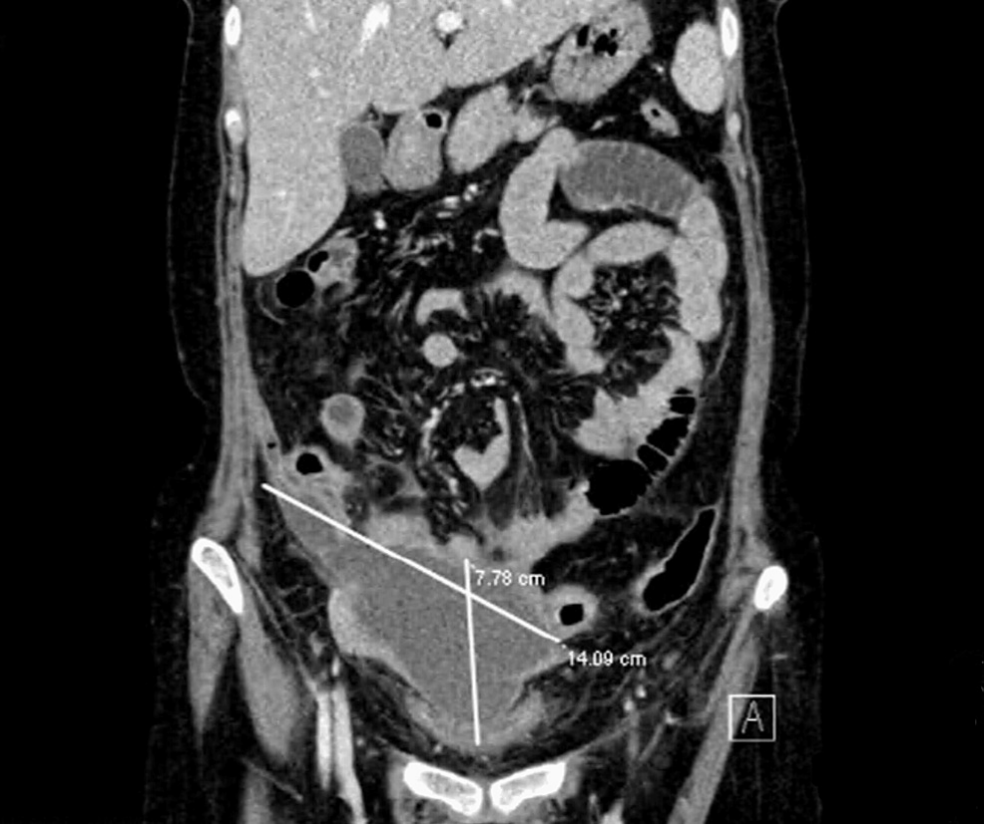
CT of the abdomen and pelvis showing a large irregular pelvic collection with rim enhancement

The radiological examination upon admission was as follows: the chest X-ray was normal while erect and supine abdominal X-rays showed nonspecific bowel gas distribution, no abnormally dilated bowel loops, and no air-fluid level. Computed tomography (CT) of the abdomen and pelvis showed no evidence of bowel ischemia, but there was a large irregular pelvic fluid collection with rim enhancement and multiple internal gas locules and surrounding fat stranding, collectively measuring 14 x 7.5 x 5.5 cm, as shown in Figure 1. The collection was seen as inseparable from the uterus and ovaries and abutting the sigmoid colon on the left side, the ascending colon on the right side, and the posterior wall of the urinary bladder. A small amount of pelvic free fluid was noted with no pneumoperitoneum. These findings were most likely related to a tubo-ovarian abscess. The posterior uterine wall had a fibroid with bilateral ovarian cysts.

The patient was admitted to the female surgical ward through the emergency department as a case of an abdominopelvic collection; tubo-ovarian abscess vs ruptured appendicitis was queried. The patient was seen by the general surgery team; they ruled out bowel perforation and appendicitis. After reviewing the abdominopelvic CT images, the source of the abdominopelvic collection was proposed to be due to bilateral TOA. Infectious diseases, nephrology, and interventional radiology were consulted regarding antibiotics choice, dialysis, and possible percutaneous drainage, respectively. She was started on triple antibiotics: metronidazole 500 mg IV three times daily, ceftriaxone 2 g IV once daily, and doxycycline 100 mg orally twice daily.

**Figure 2:**
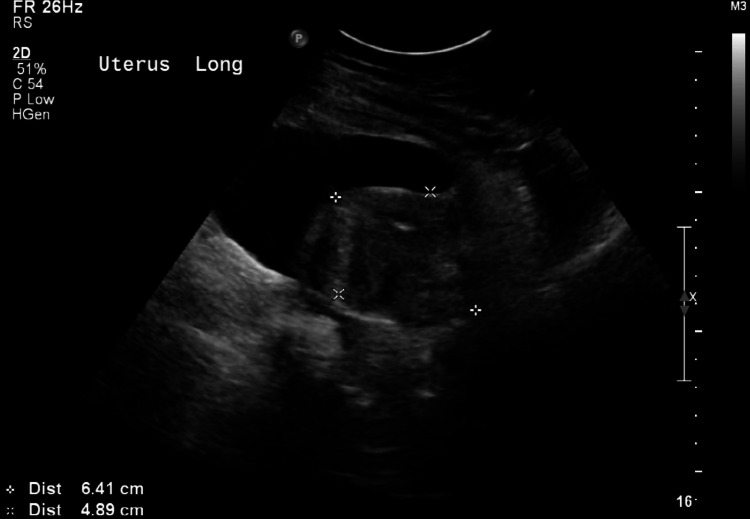
Transabdominal ultrasonography of the pelvis showing pelvic collection anterior to the uterus

Four days after starting antibiotics therapy, she had mild tenderness and a palpable smaller mass in the right lower quadrant. Transabdominal ultrasonography of the abdomen and pelvis redemonstrated the pelvic collection anterior to the uterus measuring 10 x 5 cm as shown in Figure 2. CT-guided drainage of the fluid collection was done, and a drain was inserted. Fluid analysis was positive for vancomycin-resistant *Enterococcus faecium*. Antibiotic sensitivity reported resistance to ampicillin, teicoplanin, and vancomycin but sensitivity to linezolid. The antibiotic regimen was changed according to the sensitivity pattern, to metronidazole 500 mg every eight hours and linezolid 600 mg twice daily on day 23 of admission. After two days, linezolid was switched to daptomycin 400 mg due to thrombocytopenia for the rest of the course.

**Figure 3:**
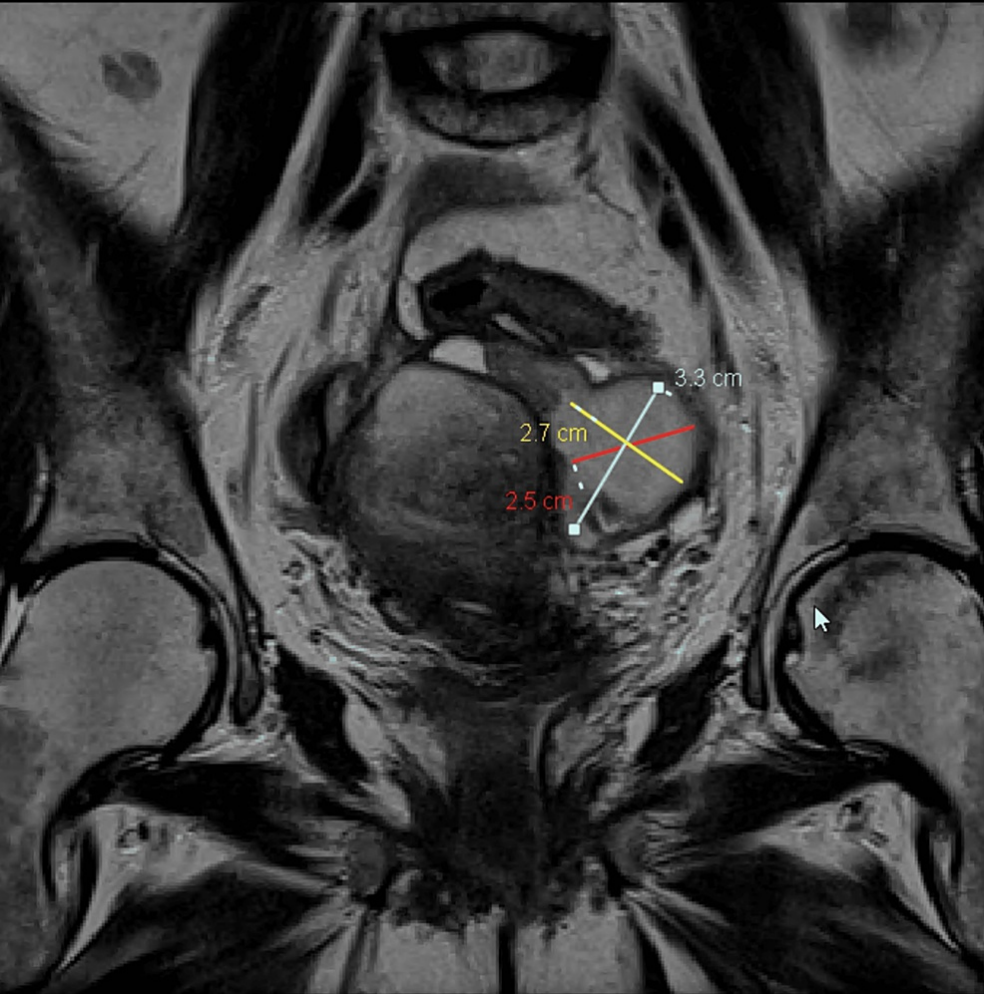
MRI of the pelvis showing the left ovarian collection

Magnetic resonance imaging (MRI) of the abdomen and pelvis on day 1 post-drain insertion showed interval drainage of the right adnexal collection, with the left ovarian collection remaining unchanged, measuring 3.3 x 2.5 x 2.7 cm shown in Figure 3. Residual inflammatory changes in the pelvis were seen in the form of soft tissue stranding and adhesion bands. The patient's clinical status improved after the percutaneous drainage. The drain output on day 1 was 200 ml thick, bloody, and purulent material. It decreased to 20 ml on day 5. The clinical exam improved and the abdomen was soft, lax, and with no tenderness.

**Figure 4:**
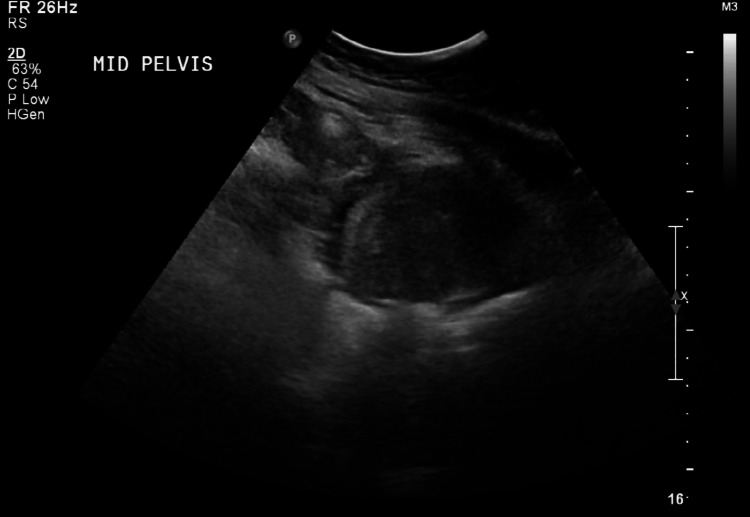
Transabdominal ultrasonography of the pelvis showing a small left adnexal collection

The last ultrasound before the patient was discharged showed no right adnexal collection, a small left adnexal fluid collection measuring 2 x 3 cm shown in Figure 4, and a single sub-serosal myoma measuring 3 x 3.5 cm. During the same admission, the patient had a right AV graft occlusion and malfunction, which was managed by a temporary central line insertion. The central line was then replaced by a permcath to continue dialysis and antibiotics administration.

The patient got discharged with the plan to continue 400 mg of IV daptomycin every other day for 14 days. As the remnant collection was small (2 x 3 cm), re-drainage wasn't necessary. She was to repeat the abdominal ultrasound one week later to monitor the pelvic collection. Regarding her endometriosis, she was prescribed dienogest 2 mg for three months with a follow-up in the gynecology clinic. She was scheduled for a follow-up in the vascular clinic regarding the occluded AV graft. The patient was seen two weeks later at the outpatient gynecology clinic, and she was in stable condition after completing the course of antibiotics. Ultrasound examination was performed, which showed regression of the pelvic collection to an unmeasurable size and the patient requested to continue her follow-up at a different hospital.

## Discussion

TOA is an infectious complex involving one of the adnexa and sometimes both. Fifteen percent (15%) of PID cases develop TOA, and 33% require admission [5]. Multiple sexual partners and previous history of PID are one of the most common predisposing factors to TOA, of which none is present in our case [2]. The commonly isolated organisms cultured from a TOA includes polymicrobial infection with a higher prevalence of *Escherichia coli* and *Bacteroides *species, of which none were isolated from our patient’s culture [2].

Based on the history given by the patient, the vague clinical presentation, and the initial biochemical markers, an initial impression of ruptured appendicitis was made. Transvaginal ultrasound is the initial imaging modality of choice to diagnose TOA, due to its convenience and cost-effectiveness. However, in our case, transvaginal ultrasound couldn’t be performed, as our patient is refusing any per-vaginal approach. For that, the modality was replaced by a transabdominal ultrasound followed by an abdominopelvic CT scan [2].

The approach to manage TOA focuses on preserving both the tubal and ovarian function to maintain the patient’s fertility for those who desire it [5]. Antibiotic therapy is the initial line of management in TOA, in addition to that drainage might be appropriate in some cases [5]. In a study on women with less than 10 cm TOA, they were blindly randomized into two groups. One group is to be treated with antibiotics alone, and the other by transvaginal aspiration combined with antibiotics. Women who underwent drainage had a shorter average hospital stay compared to the group that received only antibiotics and they were less likely to require surgical intervention [5]. In our case, transvaginal aspiration was not an option, so it was replaced by trans-abdominal drainage done by our collogues in interventional radiology with a drain kept in place to clear the area from remaining pus.

The initial antibiotic choice was made based on the Center for Disease Control and Prevention (CDC) treatment guideline, which recommends IV antibiotics for at least 24 hours with cefotetan, cefoxitin, or ceftriaxone, with or without metronidazole. Doxycycline should be administered orally, when possible, and for patients with penicillin allergy, gentamicin or clindamycin are recommended [6]. If patients fail to respond to the above regimen of IV antibiotics within 48-72 hours, the option of drainage or surgery should be considered [5]. An abscess with a diameter of 7 cm or more necessitates the intervention to drain the collection [1]. Drainage may be accomplished under the guidance of a CT or an ultrasound through the vagina, abdomen, rectum, or gluteus muscle [5]. Transvaginal drainage of the abscess plus proper antibiotics has a success rate of 90-93% and contributes to the avoidance of surgery and major complications associated with the other alternative procedures that might lead to the removal of the reproductive organs [5]. Patients who can’t be successfully treated with antibiotics and with one or more of the following, ruptured abscesses, suspected sepsis, or postmenopausal, are a candidate for surgical intervention [7]. Postmenopausal women are given the option of surgical removal of the ovary with possible bilateral salpingo-oophorectomy and hysterectomy if malignancy was suspected.

## Conclusions

The diagnosis of bilateral TOA caused by *Enterococcus faecalis* in sexually inactive women is rare. CT helps in ruling out other surgical differentials and aids in assessing the extent and size of the abscess. Treatment of the abscess should be initiated as soon as possible with broad-spectrum antibiotics as recommended by the CDC and drainage of the pelvic collection if clinically indicated. The management of TOA requires the collaboration of the gynecology, infectious diseases, and interventional radiology teams as suggested by the clinical progression of the case.

## References

[1] (2022). Epidemiology, clinical manifestations, and diagnosis of tubo-ovarian abscess. https://www.uptodate.com/contents/epidemiology-clinical-manifestations-and-diagnosis-of-tubo-ovarian-abscess.

[2] Rakheja R, Makis W, Hickeson M (2011). Bilateral tubo-ovarian abscess mimics ovarian cancer on MRI and (18)F-FDG PET/CT. Nucl Med Mol Imaging.

[3] Landers DV, Sweet RL (1983). Tubo-ovarian abscess: contemporary approach to management. Rev Infect Dis.

[4] Tokumaru T, Shima Y, Okabayashi T, Hayashi K, Yamamoto Y, Ozaki K, Iwata J (2015). Emergency surgery for tubo-ovarian abscess identified extended-spectrum beta-lactamase-producing Escherichia coli: the first case presentation revealing causative bacteria. Surg Case Rep.

[5] (2022). Management of bilateral tubo-ovarian abscesses. https://exxcellence.org/list-of-pearls/management-of-bilateral-tubo-ovarian-abscesses/.

[6] (2022). Pelvic inflammatory disease (PID). https://www.cdc.gov/std/treatment-guidelines/pid.htm.

[7] Beigi RH (2022). Management and complications of tubo-ovarian abscess. UpToDate.

